# Acoustic Source Localisation of Crack Initiation During Laser-Based DED: Experimental Validation and Challenges

**DOI:** 10.3390/ma19101967

**Published:** 2026-05-10

**Authors:** Md Jonaet Ansari, Elias J. G. Arcondoulis, Anthony Roccisano, Christiane Schulz, Thomas Schläfer, Colin Hall

**Affiliations:** 1 Future Industries Institute, Adelaide University, Mawson Lakes, SA 5095, Australiacolin.hall@adelaide.edu.au (C.H.); 2 The Australian Research Council (ARC), Industrial Transformation Training Centre in Surface Engineering for Advanced Materials (SEAM), Hawthorn, VIC 3122, Australia; 3 Institute of Sound and Vibration Research, University of Southampton, Southampton, Hampshire SO17 1BJ, UK; 4 LaserBond Ltd., Cavan, SA 5094, Australia

**Keywords:** directed energy deposition, acoustic source localisation, acoustic emission monitoring, crack detection, time difference of arrival

## Abstract

This study evaluates the feasibility of airborne acoustic source localisation (ASL) for in situ crack localisation in industrial laser-based directed energy deposition (DED-LB/M) fabricated structures. A four-microphone array combined with a Generalised Cross-Correlation with Phase Transform (GCC-PHAT) algorithm was used to estimate crack positions from time differences of arrival (TDOAs) extracted from raw acoustic emissions during multi-layer single-track fabrication. Prior to experimentation, the microphone array geometry was numerically optimised under industrial placement constraints by introducing controlled TDOA perturbations and minimising three-dimensional localisation uncertainty using alpha-shape volume analysis. Experimental validation was performed on six-layer single-track structures, with estimated crack positions compared against post-process microscopic measurements. Localisation errors ranged from 12 to 68 mm in the X-direction, 0.7–32 mm in the Y-direction, and 5–100 mm in the Z-direction. While horizontal localisation demonstrated centimetre-scale accuracy for most cracks, depth estimation exhibited greater variability. The results confirm that airborne ASL can provide meaningful spatial information regarding crack formation during DED-LB/M. However, localisation performance remains sensitive to TDOA estimation accuracy, microphone array constraints, and the complex acoustic environment inherent to the process. This work demonstrates the industrial feasibility of ASL for in situ crack investigation while highlighting the need for further advancements in array design and signal processing to achieve robust three-dimensional defect localisation in additive manufacturing systems.

## 1. Introduction

Laser-based directed energy deposition (DED-LB/M) has become an increasingly important additive manufacturing (AM) technology for the fabrication, repair, and refurbishment of high-value metallic components [1,2]. Its ability to deposit material layer by layer with precise control over geometry and composition makes it particularly attractive for aerospace, mining, defence, and energy applications [3,4]. However, the process involves complex thermo-mechanical phenomena, including rapid heating and cooling cycles, steep thermal gradients, and repeated re-melting of previously deposited layers. These conditions generate significant residual stresses, which can lead to process-induced defects such as porosity, lack of fusion, keyhole pores, delamination, and cracking [5,6]. Among these, cracks are especially critical, as they directly compromise structural integrity and may propagate under service conditions, resulting in premature failure. Consequently, reliable in situ crack detection and localisation remain key challenges for ensuring quality assurance in DED-LB/M-fabricated structures.

Acoustic emission (AE) monitoring has emerged as a promising non-contact and real-time sensing technique for detecting defects in the metal AM process [7]. When a crack initiates or propagates, it releases stored elastic energy in the form of transient stress waves, which can be captured by microphones or piezoelectric sensors [8]. Numerous studies have demonstrated that these acoustic signals contain valuable information across a broad frequency spectrum. High-frequency AE bands (hundreds of kHz to MHz) have been associated with microstructural events, while several researchers have shown that the audible frequency range (20 Hz–20 kHz) also carries meaningful information related to process instabilities and crack formation [9,10,11].

For example, Kim et al. [12] identified distinctive crack-related acoustic signatures within the 12–16 kHz range and demonstrated the feasibility of detecting both in-process and delayed cracking events. Weber et al. [13] reported frequency peaks between 11 and 18 kHz corresponding to crack and delamination defects, while Wu et al. [14] successfully correlated audible acoustic features with balling defects. Although these studies confirmed that acoustic signals can indicate defect occurrence, they primarily relied on single-sensor measurements and focused on detection rather than spatial localisation of defects.

With the advancement of data-driven approaches, machine learning (ML) techniques have been increasingly integrated into acoustic monitoring frameworks [15,16]. Chen et al. [10] developed a deep learning-based crack and keyhole pore detection framework by extracting Mel-Frequency Cepstral Coefficients (MFCCs) from denoised acoustic signals and training a convolutional neural network (CNN). Their model achieved high prediction accuracies, demonstrating the potential of ML-enhanced acoustic monitoring. However, their method predicts entire 500 ms intervals as defective if any defect is present within that region. This can lead to over-classification and does not provide insight into how many cracks or pores are present within the structure, nor does it determine their precise spatial locations. As such, while ML-based classification improves defect detection reliability, it does not address the critical need for defect localisation.

In practical industrial environments, acoustic monitoring faces additional challenges due to complex noise conditions. The DED-LB/M process generates a mixture of signals originating from laser–material interaction, powder flow, shielding gas, robotic motion, and surrounding machinery. These sources often overlap in frequency with crack-related emissions, particularly within the 0.5–6 kHz range, complicating defect identification [17,18]. Although signal denoising techniques and multi-sensor fusion frameworks have been proposed, most have been validated in controlled laboratory environments, limiting their demonstrated applicability to real-world industrial settings.

Acoustic source localisation (ASL) offers a potential solution to this limitation by estimating the spatial origin of acoustic events using arrays of sensors [19,20]. ASL techniques typically rely on time differences of arrival (TDOAs) between multiple microphones. By applying signal-processing algorithms such as the Generalised Cross-Correlation with Phase Transform (GCC-PHAT), the time delays between microphone pairs can be estimated and used to triangulate the acoustic source position [21,22]. This approach enables the determination of their spatial coordinates within the fabricated structure.

Weber et al. [23] proposed a preliminary approach for locating airborne acoustic sources using a six-microphone array in a controlled laboratory environment. Simulated acoustic events were generated using an omnidirectional speaker emitting signals around 12 kHz. Using a TDOA-based localisation method, they achieved localisation errors as low as 3.9 mm under favourable conditions, although errors up to 75 mm were also reported. While this study demonstrated the theoretical feasibility of airborne acoustic localisation, it relied on simulated tonal signals rather than real crack emissions and did not validate the approach during actual DED-LB/M fabrication.

To date, the application of multi-microphone ASL systems for localising real crack events in DED-LB/M-fabricated structures remains largely unexplored, particularly under industrial conditions characterised by high noise levels, complex geometries, and dynamic process environments. Furthermore, the localisation of cracks in three-dimensional, multi-layered builds presents additional challenges due to reflections, attenuation, and uncertainties in acoustic wave propagation paths.

This study addresses this critical gap by experimentally investigating the application of acoustic source localisation for crack investigation in laser-based DED processes. An array of acoustic sensors combined with the GCC-PHAT algorithm is employed to estimate crack locations using the TDOA values derived from raw acoustic recordings. The proposed approach is validated on multi-layered single-track structures with localisation results compared against post-process microscopic examination. In addition to evaluating localisation accuracy, this work systematically examines the challenges associated with signal-to-noise ratio variability, frequency overlap between crack signatures and process noise, and limitations of conventional GCC-PHAT-based algorithms in industrial environments.

By bridging the gap between acoustic defect detection and spatial localisation, this research contributes toward the development of a more comprehensive in situ monitoring framework for DED-LB/M. The findings provide critical insight into both the practical feasibility and current limitations of ASL for real-time crack investigation in industrial additive manufacturing systems.

## 2. Acoustic Source Localisation Methodology

### 2.1. Localisation Principles

Acoustic source localisation (ASL) is based on three primary geometric approaches: trilateration, triangulation, and multilateration [20,22]. Among these, multilateration is the most suitable for airborne acoustic monitoring, where neither the absolute emission time nor angular measurements are typically available [24]. This approach estimates the source position from the time difference of arrival (TDOA) between spatially separated microphones. Each TDOA measurement constrains the possible source location to a hyperboloid surface whose parameters depend on sound speed, microphone spacing, and the measured time delay. The intersection of multiple hyperboloids derived from several microphone pairs yields the acoustic source coordinates.

In this study, a four-microphone array was employed, and the crack source position was determined through numerical optimisation using the calculated TDOA values.

### 2.2. Time Difference of Arrival Estimation

Accurate estimation of the time difference of arrival (TDOA) is fundamental to reliable acoustic source localisation, as the localisation accuracy directly depends on the precision of delay estimation. In industrial directed energy deposition with laser beam melting (DED-LB/M) environments, acoustic signals are often degraded by background noise, reflections from metallic surfaces, and spectral distortions, necessitating robust delay estimation methods.

In this study, the Generalised Cross-Correlation with Phase Transform (GCC-PHAT) method was employed for TDOA estimation due to its proven robustness in noisy and reverberant conditions [25,26]. The method computes the cross-power spectrum between two microphone signals, applies a phase-only weighting to suppress amplitude variations, and identifies the time lag corresponding to the correlation peak. This phase normalisation improves estimation accuracy by mitigating the effects of reverberation and spectral distortion.

While acoustic beamforming provides high spatial resolution and the capability to isolate individual sound sources, conventional beamforming systems typically require large microphone arrays (40–50 channels) and extensive data acquisition hardware [27].These requirements increase costs and reduce mobility, making such systems impractical for field deployment or industrial environments where portability and resource efficiency are critical [28,29].

The GCC-PHAT approach, by contrast, offers a computationally efficient and cost-effective solution for TDOA-based source localisation. Its reduced hardware requirements make it particularly well-suited for implementation in DED-LB/M monitoring, where maintaining accuracy under constrained conditions is a key consideration.

## 3. Numerical Methodology and Array Optimisation

### 3.1. Mathematical Modelling

The acoustic source localisation (ASL) framework developed in this study employs a four-microphone array (n = 4) arranged in three-dimensional space. This configuration was selected to provide sufficient spatial information for robust multilateration while maintaining computational efficiency and practical feasibility for industrial deployment. The microphones, designated as Mic. #1, Mic. #2, Mic. #3, and Mic. #4, capture airborne acoustic emissions generated during the DED-LB/M process. The TDOA values are calculated relative to a reference microphone (Mic. #1).

The unknown source coordinates (x, y, z) are determined by solving the nonlinear multilateration equation [30]:

(1) $${(x_{i\;}-x)}^{2}\;+\;{(y_{i}-y)}^{2}\;+\;{(z_{i}-z)}^{2}\;={\;\left(c\cdot \left(t_{i}-t_{1}\right)\;+\;\sqrt{{\left(x_{1}-x\right)}^{2}\;+\;{\left(y_{1}-y\right)}^{2}\;+\;{\left(z_{1}-z\right)}^{2}}\right)}^{2}$$

where (*x*, *y*, *z*) is the unknown source location, (*x_i_*, *y_i_, z_i_*) are the known microphone positions, *c* is the speed of sound, and *t_i_* − *t*_1_ is the TDOA between microphone *i* and the reference microphone (Mic. #1).

**Figure 1:**
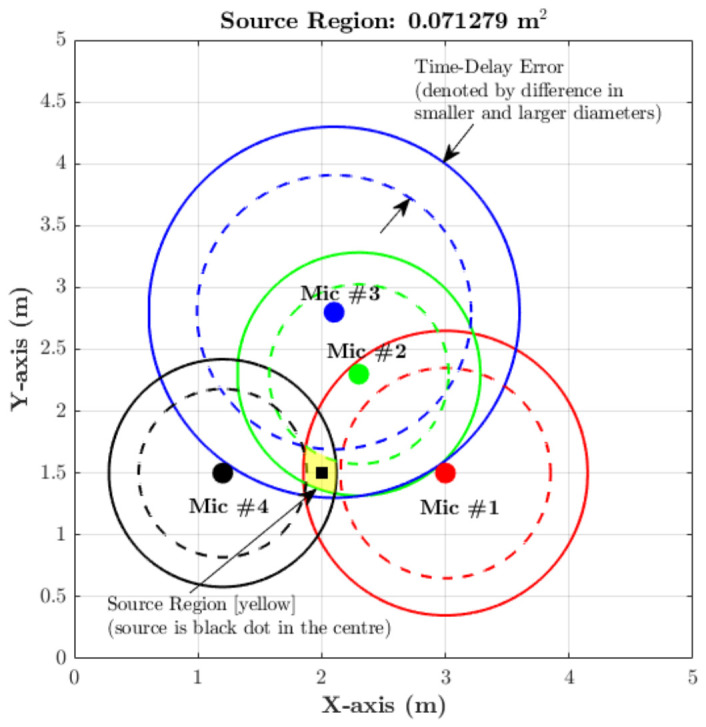
Graphical representation of source localisation with four microphones. The source region [yellow] represents the overlapping area; source is the black dot in the centre. Time-delay error is represented by the difference in radii.

The geometric interpretation of this multilateration principle is illustrated conceptually in Figure 1. In this figure, circular regions (two-dimensional analogues of spherical surfaces) are constructed around each of the four microphones. The true source location is represented by the black dot at the centre. Due to TDOA uncertainty, two radii are defined for each microphone: one slightly larger and one slightly smaller than the nominal source distance. The difference between these radii represents the time-delay error. The intersection of the larger circles defines a feasible source region, while subtraction of the smaller circles refines this region. The resulting overlapping area, highlighted in yellow, represents the estimated source region under timing uncertainty. Although Figure 1 provides a two-dimensional visualisation for clarity, the actual implementation of the localisation algorithm is performed in three-dimensional space, where the equivalent geometric entities are spherical shells, whose intersections define a bounded source volume.

Because Equation (1) is nonlinear, a numerical optimisation routine was implemented in MATLAB R2025a using the fsolve function with the Levenberg–Marquardt algorithm. This approach iteratively refines the estimated source coordinates by minimising the residual between the measured and predicted TDOA values. All available TDOA measurements are processed simultaneously to produce a single optimised three-dimensional source estimate.

Given that the intended application is within an industrial DED-LB/M environment, measurement uncertainties in TDOA estimation are unavoidable due to background noise, reflections, and signal distortion. To assess the robustness of the localisation model, a numerical sensitivity analysis was conducted by introducing a 1% artificial perturbation to the calculated TDOA values. This enabled the evaluation of localisation uncertainty and informed subsequent optimisation of the microphone array configuration.

### 3.2. Microphone Array Configuration Optimisation

The optimisation of microphone array geometry is critical for improving the accuracy and robustness of acoustic source localisation in complex industrial environments such as DED-LB/M systems. In this study, a comprehensive numerical analysis was conducted to systematically explore feasible microphone arrangements and evaluate their localisation performance under realistic TDOA uncertainties.

**Figure 2:**
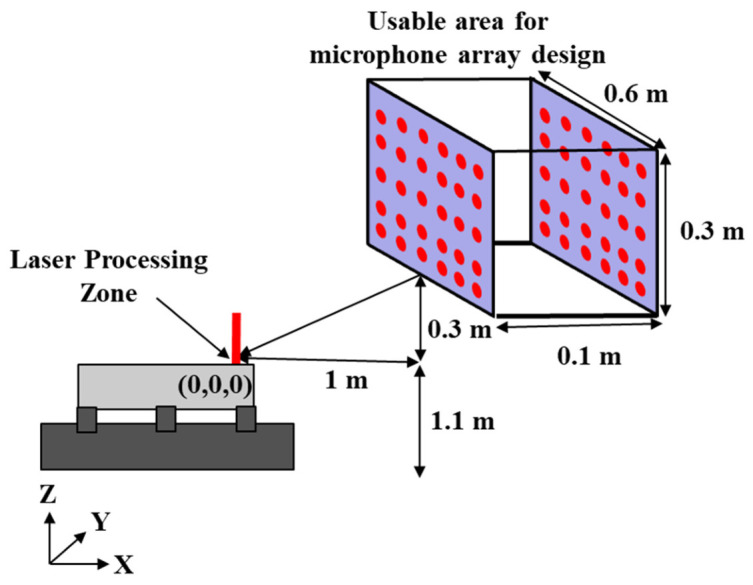
Schematic representation of the laser processing zone at the origin and the surrounding usable area for microphone array placement.

The experiments were performed in an industrial setting, where the microphone array had to be positioned safely without interfering with the laser operator or the deposition process. As illustrated in Figure 2, the laser processing zone was defined as the origin (0, 0, 0), and the surrounding usable region was identified for microphone placement. The laser head operates approximately 1.1 m above ground, and frequent movement occurs near this zone. Consequently, the allowable placement region for the microphone holder was restricted to:

- X-direction: 1.0–1.1 m.
- Y-direction: −0.3–0.3 m.
- Z-direction: 0.3–0.6 m.

This spatial constraint ensured safe operation while maintaining adequate acoustic coverage of the processing area.

For optimisation purposes, the acoustic source was assumed to be located at (0, 0, 0). Mic. #1 was fixed at (1, 0, 0.45) m to serve as the reference microphone. The remaining three microphones varied systematically within the defined spatial limits. As summarised in Table 1, Mic. #2 and Mic. #3 were constrained to X = 1 m with Y positions ranging from −0.3 to 0.3 m (in 0.1 m increments) and Z positions between 0.3 and 0.6 m. Mic. #4 was positioned at X = 1.1 m with the same Y and Z variation ranges.

**Figure 3:**
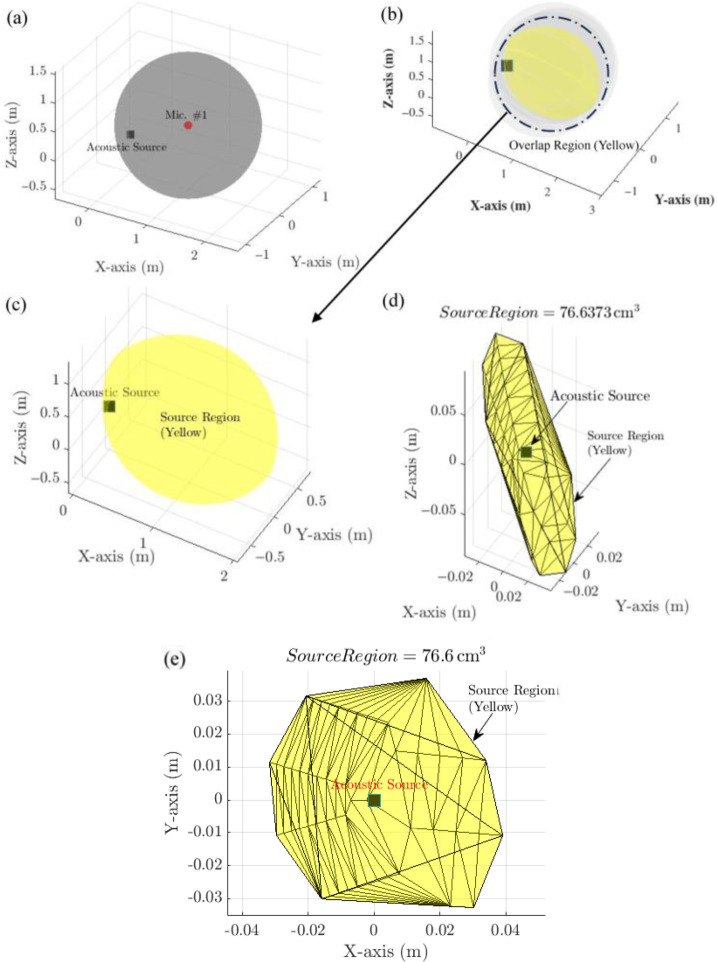
Spatial representation of acoustic source localisation. (**a**) 3D overview showing the acoustic source and microphone “Mic. #1,” with a sphere generated using larger radii of Mic. #1 to represent sound propagation. (**b**) The side view of the 3D space illustrates the spherical surface overlap region for each microphone. (**c**) Zoomed-in view of the overlap region in the X–Y plane. (**d**) Estimated source region (yellow) within the overlap area for array 1. (**e**) Estimated source region (yellow) in the X–Y plane.

The feasible microphone coordinates were generated using MATLAB’s meshgrid function and reshaped into three-dimensional coordinate arrays for efficient computation. To evaluate all possible configurations, MATLAB’s combvec function was employed to generate combinations of positions for Mic. #2, Mic. #3, and Mic. #4, while Mic. #1 remained fixed. This resulted in 21,952 possible microphone array configurations. For illustrative purposes, one representative configuration is shown in Figure 3 and defined as:

- Mic. #1: (1, 0, 0.45) m.
- Mic. #2: (1, 0.3, 0.3) m.
- Mic. #3: (1, −0.3, 0.3) m.
- Mic. #4: (1.1, −0.1, 0.6) m.

For each configuration, an iterative numerical analysis was performed. First, Euclidean distances between the source and each microphone were computed. Corresponding time delays were derived using the speed of sound (343 m/s). To simulate realistic measurement uncertainty, a 1% error was introduced to the calculated time delays. This produced two sets of radii for each microphone: one slightly larger and one slightly smaller than the nominal source distance.

Spherical surfaces corresponding to these perturbed radii were generated using MATLAB’s sphere function. Figure 3a shows a three-dimensional overview of this process, where a sphere generated from the larger radius of Mic. #1 represents the potential sound propagation region. Figure 3b presents a side view illustrating the overlap between spherical surfaces from multiple microphones. Figure 3c provides a zoomed-in view of the overlap region in the X–Y plane.

To compute the intersection volumes accurately, the spherical surfaces were converted into alpha shapes using MATLAB’s alphaShape function. This computational geometry approach enables robust representation of irregular three-dimensional intersections. The intersection of the larger spheres from all four microphones defined the initial feasible source region. Subsequently, the smaller spheres were subtracted from this region to refine the localisation estimate by eliminating areas inconsistent with the TDOA bounds. The resulting refined source region is highlighted in yellow in Figure 3d, while Figure 3e presents its projection onto the X–Y plane.

The final alpha shape for each configuration represents the bounded three-dimensional region in which the source could be located under a 1% TDOA error. These alpha shapes were stored for all 21,952 configurations for comparative evaluation.

**Figure 4:**
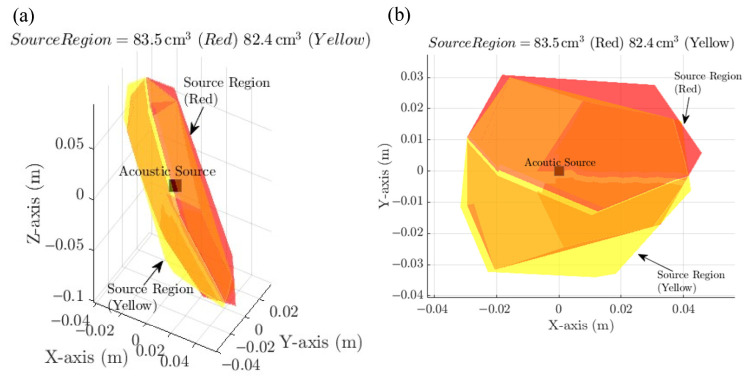
Estimated source regions for microphone Arrays 2 (yellow) and 3 (red). (**a**) 3D view in the X–Y–Z plane. (**b**) 2D projection on the X–Y plane.

Upon completion of the iterative process, each configuration was assessed based on both the volume and geometric compactness of the estimated source region. Volume was computed using MATLAB’s built-in volume function, while geometric characteristics such as aspect ratio and spatial distribution were analysed to avoid elongated or poorly constrained regions. Figure 4 illustrates example estimated source regions for Arrays 2 and 3, showing both three-dimensional (X–Y–Z) views and two-dimensional projections on the X–Y plane.

Three configurations demonstrated superior performance, as summarised in Table 2. Even with a 1% TDOA perturbation, the estimated localisation volumes were:

- Array 1: 76.7 cm^3^.
- Array 2: 82.4 cm^3^.
- Array 3: 83.5 cm^3^.

Array 1 produced the smallest and most compact source region (76.7 cm^3^), as shown in Figure 3d,e. Its microphone positions were:

- Mic. #1: (1, 0, 0.45) m.
- Mic. #2: (1, 0.3, 0.3) m.
- Mic. #3: (1, −0.3, 0.3) m.
- Mic. #4: (1.1, −0.1, 0.6) m.

This configuration was therefore selected as the optimal array geometry for experimental implementation in the DED-LB/M environment.

Prior to industrial deployment, the selected array was validated in an anechoic chamber to confirm localisation performance under reflection-free conditions. Following successful validation, the optimised array was integrated into the industrial DED-LB/M setup for crack localisation experiments.

## 4. Experimental Methodology

### 4.1. Microphone Array Configuration and Data Acquisition

The optimised microphone array configuration identified through numerical analysis was implemented experimentally using four 1/4-inch omnidirectional free-field microphones (MPA416, BSWA Technology Co., Ltd., Beijing, China). Mic. #1 was designated as the reference microphone and defined as the origin (0, 0, 0) of the local coordinate system. The remaining microphones were positioned relative to this reference point according to the optimised geometry (Array 1).

An adjustable aluminium channel mounting system was fabricated to ensure precise positioning and structural stability of the microphones during experiments. This modular structure enabled accurate alignment with the optimised coordinates while maintaining rigidity under both laboratory and industrial operating conditions.

The relative microphone coordinates were set as follows:

- Mic. #2: (0, 0.3, −0.15) m.
- Mic. #3: (0, −0.3, −0.15) m.
- Mic. #4: (0.1, −0.1, 0.15) m.

**Figure 5:**
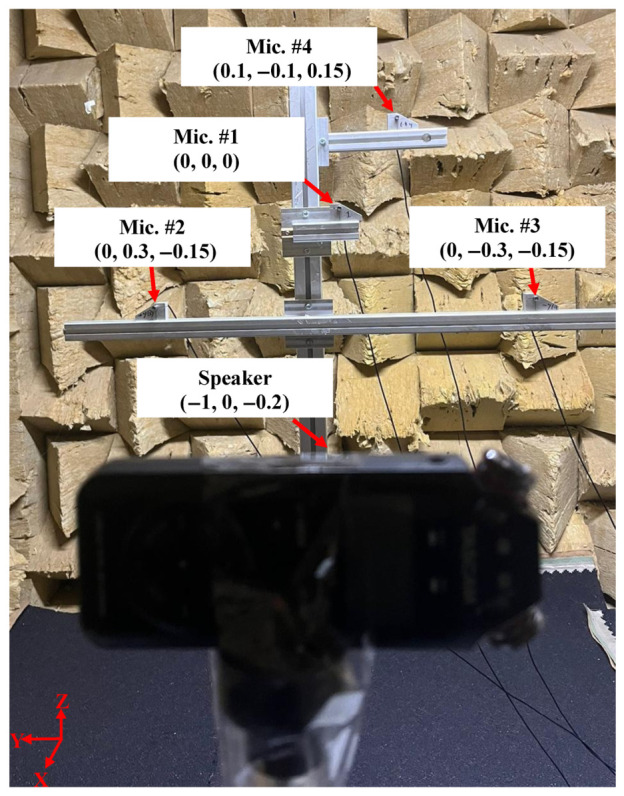
Experimental setup for microphone array error analysis in acoustic source localisation, conducted within an anechoic chamber.

The complete array geometry is illustrated in Figure 5, which shows the spatial arrangement of the four microphones and highlights the optimised configuration used for acoustic source localisation.

Acoustic signals from the microphones were recorded using a National Instruments cDAQ-9174 CompactDAQ chassis (National Instruments Corporation, Austin, TX, USA), a four-slot USB-based platform capable of synchronous multi-channel acquisition. The selection of hardware was guided by practical considerations, including cost efficiency, portability, and suitability for industrial deployment. A custom-developed MATLAB interface was implemented to communicate with the NI DAQ system, enabling real-time acquisition, storage, and subsequent signal processing for TDOA estimation and source localisation.

### 4.2. Anechoic Chamber Validation and Error Analysis

Prior to deployment in the industrial DED-LB/M environment, the microphone array and localisation algorithm were validated under controlled acoustic conditions in an anechoic chamber located in the Computation Physics Laboratory at the University of South Australia.

The anechoic chamber is designed to eliminate sound reflections and simulate free-field conditions. Its internal dimensions are 4.75 × 3.90 × 3.94 m, and it is effectively anechoic for frequencies above 100 Hz. The walls and ceiling are lined with sound-absorbing wedges measuring 100 mm × 100 mm at the base and 300 mm in length. The floor consists of an open aluminium lattice structure with thick acoustic foam installed beneath to minimise reflections.

The experimental setup within the chamber is shown in Figure 5, illustrating the microphone array and the controlled acoustic source used for validation. This reflection-free environment enabled independent verification of the GCC-PHAT-based TDOA estimation and multilateration algorithm without interference from environmental noise.

To quantify localisation accuracy, a controlled positional error analysis was performed. Both white noise and tonal signals were emitted from a compact audio recorder speaker (Tascam DR-05X; TEAC Corporation, Tokyo, Japan), which acted as a near point-source emitter with an approximate diameter of 10 mm. The speaker was rigidly mounted on a precision traverse to allow accurate spatial positioning.

During testing, the Z-coordinate of the acoustic source was fixed at −0.2 m relative to the reference microphone, while X- and Y-coordinates were systematically varied. Nine measurement positions were defined in the horizontal plane:

- (X = −1) m with (Y = [−0.1:0.05:0.1]) m.
- (Y = 0) m with (X = [−0.9:0.05:−1.1]) m.
- (Z = −0.2) m.

This structured grid enabled comprehensive evaluation of localisation performance across different lateral positions.

**Figure 6:**
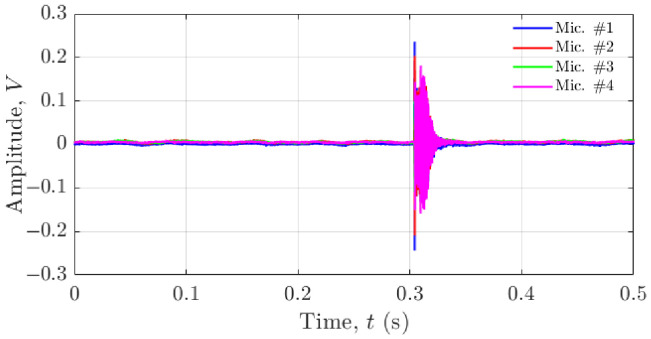
Voltage of acoustic far-field pressures over time, illustrating the temporal variation in the signal emitted from the speaker positioned at X = −1 m, Y = 0 m, Z = −0.2 m.

Figure 6 presents an example of the recorded far-field acoustic pressure (voltage) over a 0.5 s interval for a tonal signal emitted from the source located at (−1, 0, −0.2) m. These raw signals were processed using the GCC-PHAT algorithm to estimate TDOA values between microphone pairs (TDOA_12_, TDOA_13_, and TDOA_14_).

**Figure 7:**
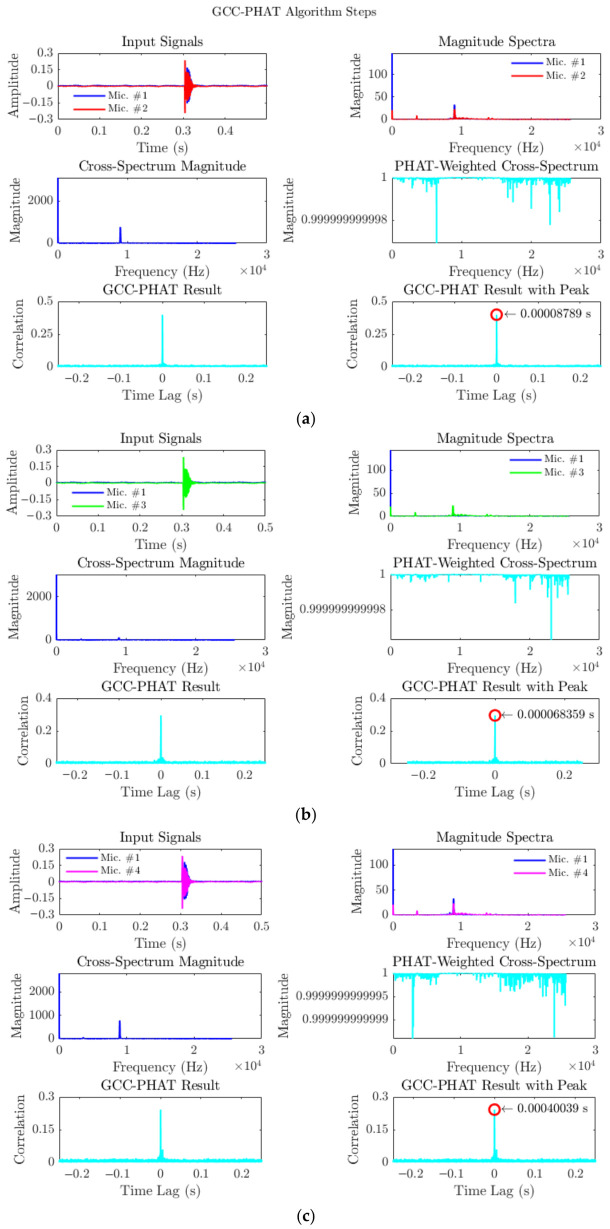
Step-by-step illustration of the GCC-PHAT function process for estimating TDOA values shows the process for (**a**) TDOA_12_ (Mic. #1 and Mic. #2), (**b**) TDOA_13_ (Mic. #1 and Mic. #3), and (**c**) TDOA_14_ (Mic. #1 and Mic. #4).

The complete TDOA estimation procedure is illustrated in Figure 7, which shows step-by-step GCC-PHAT outputs for each microphone pair. For example, the estimated time delays were:

- TDOA_12_ = 0.000225 s.
- TDOA_13_ = −0.0000878 s.
- TDOA_14_ = 0.000341 s.

These TDOA values were then used as inputs to the nonlinear optimisation routine (Equation (1)) to compute the three-dimensional source location.

**Figure 8:**
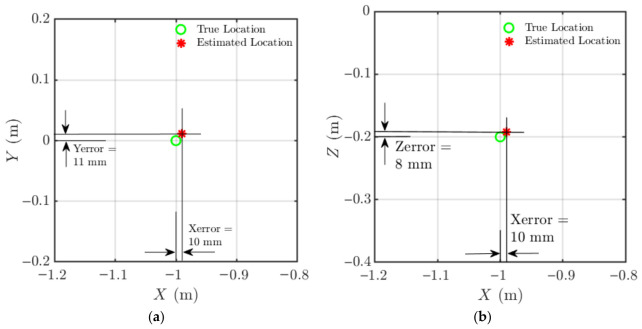
Acoustic source localisation results in (**a**) the X–Y plane and (**b**) the X–Z plane for an acoustic source located at X = 1 m, Y = 0 m, Z = −0.2 m. True source position (green circle) and estimated source location (red asterisk) are shown for both planes. Lines at the identified source location highlight the estimation error relative to the true source.

Localisation results for the example case are shown in Figure 8, where the true source position (green circle) and the estimated position (red asterisk) are plotted in both the X–Y and X–Z planes. The resulting localisation errors were:

- X-direction: 10 mm.
- Y-direction: 11 mm.
- Z-direction: 10 mm.

**Figure 9:**
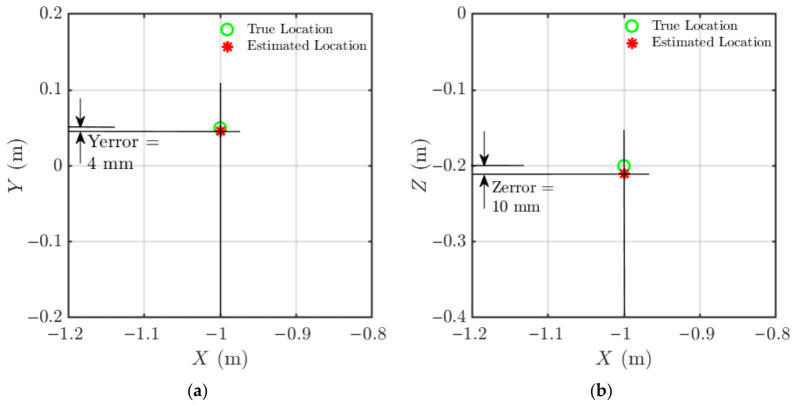
Acoustic source localisation results in (**a**) X–Y plane and (**b**) X–Z plane for an acoustic source located at X = 1 m, Y = 0.05 m, Z = −0.2 m.

The second example is presented in Figure 9 for a source located at (−1, 0.05, −0.2) m. The corresponding errors were 0 mm in X, 4 mm in Y, and 10 mm in Z.

**Figure 10:**
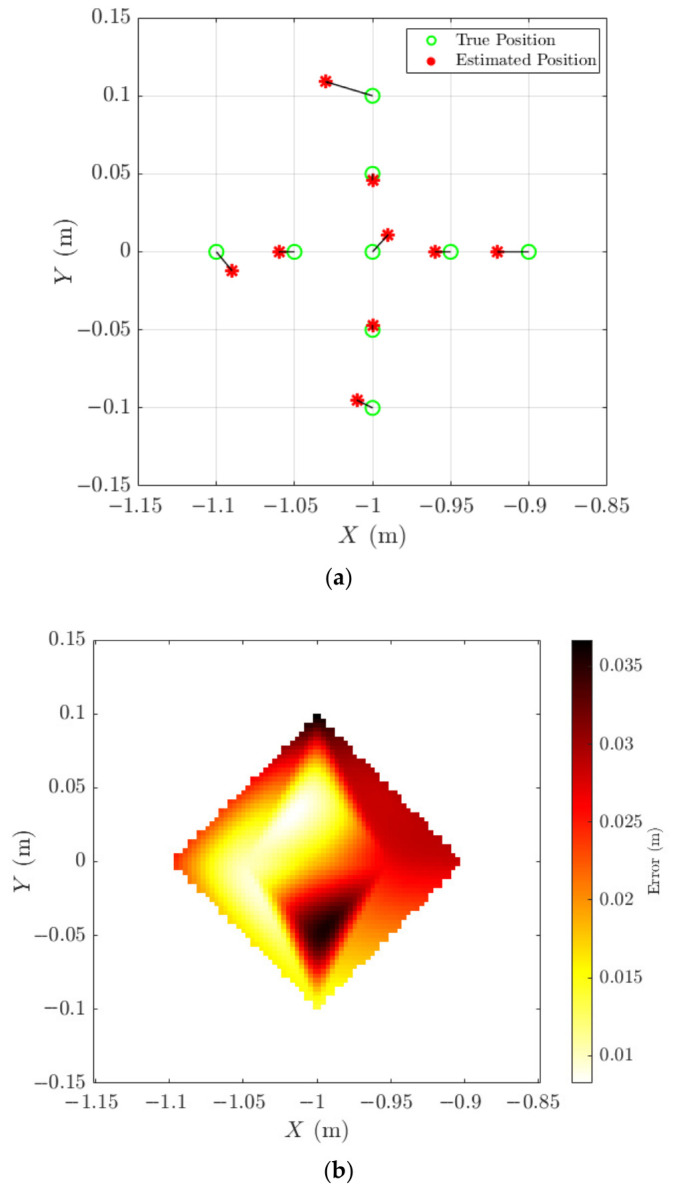
Evaluation of the ASL system’s performance. (**a**) Comparison of true source positions (green circles) and estimated source locations (red asterisks) in the X–Y plane. (**b**) Colour-coded error map illustrating the ASL system’s localisation accuracy for acoustic sources across various positions in the X–Y plane.

Figure 10a summarises localisation performance across all nine measurement positions in the X–Y plane. True positions (green circles) and estimated positions (red asterisks) demonstrate close agreement. X-direction errors ranged from 0 to 30 mm, with the largest deviation occurring when the true position was −1 m and estimated at −1.03 m. Y-direction errors ranged between 1 mm and 11 mm, with most estimates remaining within 5 mm of the true position. Figure 10b provides a colour-coded error map illustrating spatial variation in localisation error across the horizontal plane, confirming consistent performance within ±30 mm in the tested region.

**Figure 11:**
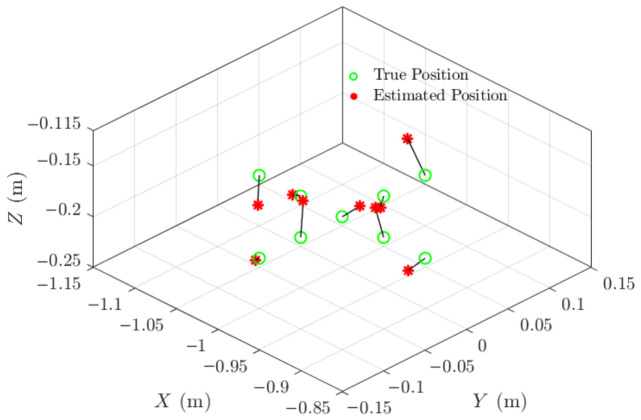
Comparison of true source positions (green circles) and estimated source locations (red asterisks) in 3D space.

Three-dimensional localisation performance is shown in Figure 11, where true and estimated source positions are plotted in 3D space. Although the Z-coordinate was fixed at −0.2 m during testing, Z-direction errors ranged from 3 mm to 35 mm, with an average error of approximately 15 mm.

Overall, the anechoic chamber validation demonstrated strong localisation accuracy, particularly in the horizontal plane, confirming the reliability of the GCC-PHAT-based multilateration framework prior to its application in the industrial DED-LB/M environment.

### 4.3. Fabrication of DED-LB/M Structures

An industrial DED-LB/M system was employed to develop a setup and protocol to pinpoint crack locations in DED-LB/M-fabricated structures. The system was equipped with four fibre-coupled near-infrared diode laser sources, providing a maximum combined power capacity of 16 kW. A commercially sourced nickel-based alloy reinforced with tungsten carbide hard particles was selected as the deposition material. The material was deposited onto a flat mild steel substrate, which served as the baseplate for fabrication. The experimental trials were conducted using specific process parameters, as detailed in Table 3. The fabrication procedure involved the production of two single tracks, each comprising multiple layers, deposited using a unidirectional laser scanning pattern. This approach was designed to simulate typical DED-LB/M built structures while potentially inducing thermal stresses that could lead to crack formation.

**Figure 12:**
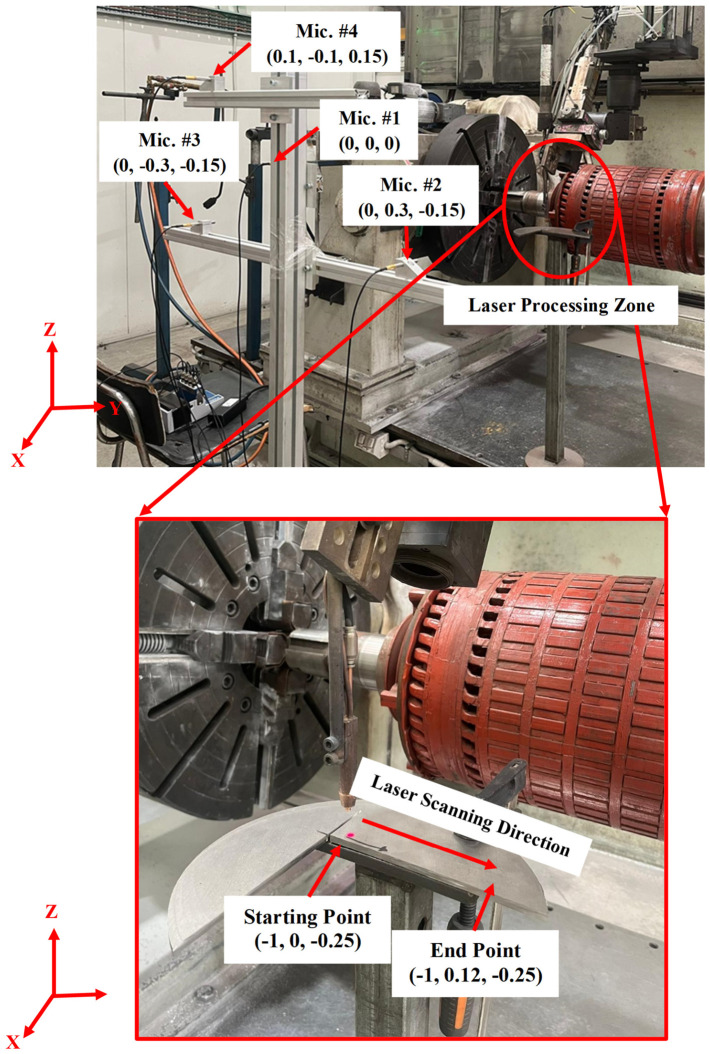
(**Top**): Experimental setup for the real-time capture of acoustic signals using the microphone array during the DED-LB/M process. (**Bottom**): Zoomed-in view of the laser processing zone, illustrating the laser scanning direction from left to right.

Figure 12 illustrates the experimental setup, which was specifically arranged to enable real-time capture of acoustic signals during the DED-LB/M process. This setup incorporated a microphone array positioned strategically to detect and record acoustic emissions that might indicate crack formation or propagation.

Upon completion of the DED-LB/M fabrication process, the produced tracks were allowed to cool naturally under ambient conditions until thermal equilibrium was reached. A dye penetrant inspection was subsequently performed on the fabricated specimens to identify and quantify surface-breaking cracks along the deposited tracks.

Following surface cleaning, high-resolution topographical images were obtained using an Olympus DSX1000 digital optical microscope (Olympus Corporation, Hachioji, Tokyo, Japan). These surface images enabled precise identification of crack positions and measurement of the distances between individual cracks. The experimentally measured crack coordinates were then treated as ground-truth reference locations and compared with the acoustically estimated positions to quantify the localisation error of the ASL system.

## 5. Results and Discussion

The acoustic signals recorded during fabrication of Track 1 and Track 2 were analysed using the GCC-PHAT-based multilateration framework described previously. The objective was to detect and spatially localise crack events within DED-LB/M-fabricated structures under real processing conditions.

### 5.1. Crack Localisation in Multi-Layer Single Tracks

Both Track 1 and Track 2 consisted of six deposited layers. Crack localisation analysis focused on the 6th layer, where thermal accumulation and residual stress were most pronounced and crack occurrence was observed to increase.

#### 5.1.1. Track 1

**Figure 13:**
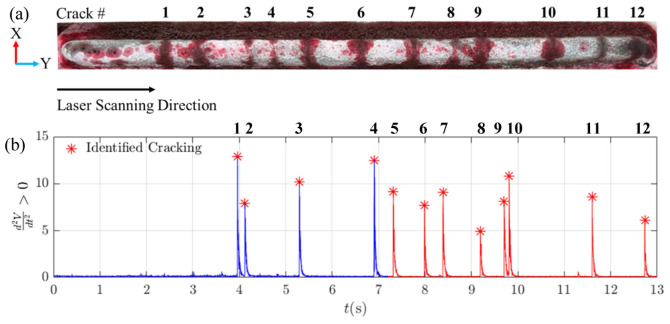
Crack detection in DED-LB/M-fabricated Track 1. (**a**) Dye penetrant test results on the as-cladded sample, with crack marked by numbers. (**b**) Processed acoustic signal from the 6th layer of Track 1, showing identified cracking events marked by red asterisks.

Figure 13b presents the processed acoustic signal recorded from Mic. #1 during the fabrication of the 6th layer of Track 1. The signal is colour-coded to indicate process states:

- Blue regions represent active laser deposition.
- Red regions represent laser-off periods.

Twelve crack events were identified during and immediately after deposition of the 6th layer. The occurrence times of these cracks, denoted as (*t_c_*), were recorded at 3.96 s, 4.12 s, 5.29 s, 6.90 s, 7.31 s, 7.99 s, 8.38 s, 9.19 s, 9.70 s, 9.81 s, 11.6 s, and 12.73 s and are marked by red asterisks in Figure 13b.

To validate the acoustic detection results, dye penetrant testing was conducted. Figure 13a shows the as-cladded surface with crack positions labelled numerically. The number and distribution of cracks identified via dye testing were consistent with the acoustically detected events, confirming the reliability of the detection stage.

The true crack locations were determined using high-resolution top surface imaging with an Olympus DSX1000 digital microscope (Olympus Corporation, Hachioji, Tokyo, Japan). Because the track was fabricated along with the positive Y-direction while X and Z remained fixed, crack positions were expressed as:

(−1, *Y_i_*, −0.25)

where (*Y_i_*) corresponds to the measured distance from the start of the track. These true coordinates are listed in Table 4.

**Figure 14:**
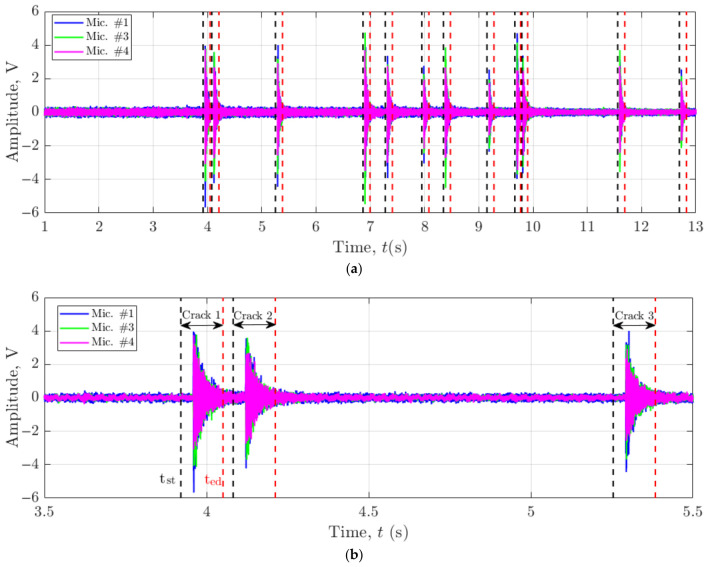
Acoustic signal analysis of crack events during DED-LB/M fabrication. (**a**) Time-windowed raw acoustic signals from three microphones captured during the fabrication of Track 1 (6th layer). (**b**) Magnified view of the raw acoustic signal from time frame 3.5 s to 5.5 s, demonstrating that the selected time window successfully captured the complete acoustic profile of the crack events.

To determine each crack’s precise location within the DED-LB/M-fabricated structures, a time window was defined around each crack’s occurrence time to extract relevant data segments. This analysis utilised acoustic signals from three microphones: Mic. #1, Mic. #3, and Mic. #4. It is important to note that Mic. #2 was inactive during the fabrication of both Track 1 and Track 2, necessitating the use of data from only three microphones for this localisation process. In this configuration, three microphones provide the minimum number of TDOA measurements required for three-dimensional multilateration; however, this reduces the redundancy that was built into the numerically optimised four-microphone array and increases the sensitivity of the solution to TDOA noise, particularly in the Z-direction where the effective array aperture is the smallest. Consequently, the depth estimates are more susceptible to small timing errors than the in-plane (X–Y) localisation, and reactivating Mic. #2 or adding additional vertically separated sensors is expected to reduce this uncertainty and improve robustness. This approach allowed for the isolation and analysis of specific acoustic events associated with crack formation. The start time (*t_st_*) for each crack was calculated by subtracting 0.04 s from the crack occurrence time (*t_c_*), while the end time (*t_ed_*) was determined by adding 0.09 s to *t_c_*. The start and end times of the data window were marked with black and red dashes, as shown in Figure 14a. This approach resulted in a 0.13 s data window for each crack event. To provide a clearer visualisation of the acoustic profile, Figure 14b presents a magnified view of the raw acoustic signal from the time frame 3.5 s to 5.5 s. This zoomed-in perspective demonstrates that the selected time window successfully captured the complete acoustic signature of the crack events, allowing for detailed analysis of the signal characteristics associated with each crack occurrence.

Subsequently, the GCC-PHAT algorithm was employed to determine the TDOA values between signals received at different microphones: Mic. #1 and Mic. #3 (TDOA_13_), and Mic. #1 and Mic. #4 (TDOA_14_), for individual crack events.

**Figure 15:**
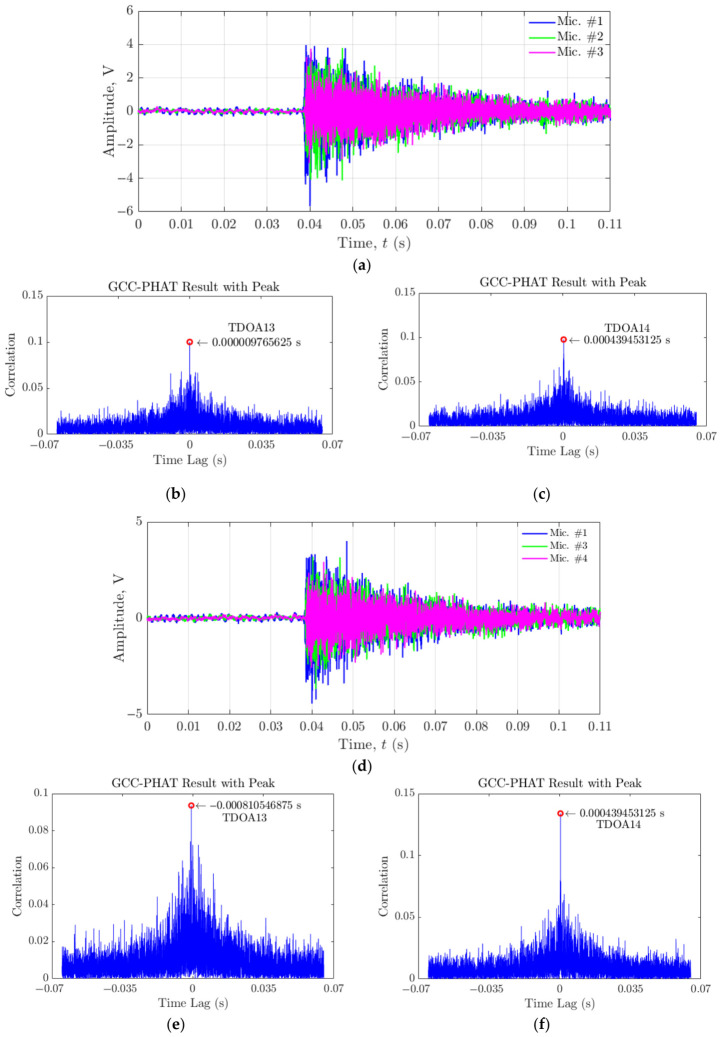
Time-windowed acoustic signals and TDOA estimation for cracks in DED-LB/M-fabricated structures. (**a**) Raw acoustic signals from three microphones for Crack 1. Estimated TDOA values using the GCC-PHAT algorithm for (**b**) Mic. #1 and Mic. #3 (TDOA_13_), and (**c**) Mic. #1 and Mic. #4 (TDOA_14_) for Crack 1. (**d**) Raw acoustic signals from three microphones for Crack 3. (**d**) Estimated TDOA values using the GCC-PHAT algorithm for (**e**) Mic. #1 and Mic. #3 (TDOA_13_), (**f**) Mic. #1 and Mic. #4 (TDOA_14_) for Crack 3.

Figure 15 demonstrates the time-windowed acoustic signals and TDOA estimation for Cracks 1 and 3 as examples. For cracks 1 and 3, the TDOA values were estimated as shown in Table 5. The table presents the TDOA_13_ and TDOA_14_ values for these two cracks, calculated using Mic. #1 and Mic. #3, and Mic. #1 and Mic. #4, respectively. A similar procedure was applied to calculate TDOA values for all 12 cracks. Using these TDOA values, the estimated crack locations were then calculated.

The results of these crack source localisations in X-, Y-, and Z-coordinates were systematically saved in an array called *CSL_all_*. The estimated position of each crack was determined as shown in Table 6.

The analysis of the results comparing the true crack positions with the estimated crack positions reveals both the capabilities and limitations of the ASL technique in localising cracks in DED-LB/M-fabricated structures. In the X-direction, the technique showed errors ranging from 12 mm to 32 mm. The Y-direction estimations exhibited a more refined accuracy, with errors spanning from 4 mm to 27 mm for most cracks. However, the Z-direction estimates displayed a wider range of errors, varying between 5 mm and 100 mm for the detectable cracks. For the present geometry, source–microphone distances are on the order of 1 m, corresponding to nominal propagation times of approximately 2.9 ms at 343 m/s. Thus, centimetre-scale localisation errors correspond to TDOA perturbations of roughly 30–90 µs, whereas the metre-scale deviations observed for Cracks 3, 8, and 11 imply accumulated TDOA errors of several hundred microseconds across the available microphone pairs.

**Figure 16:**
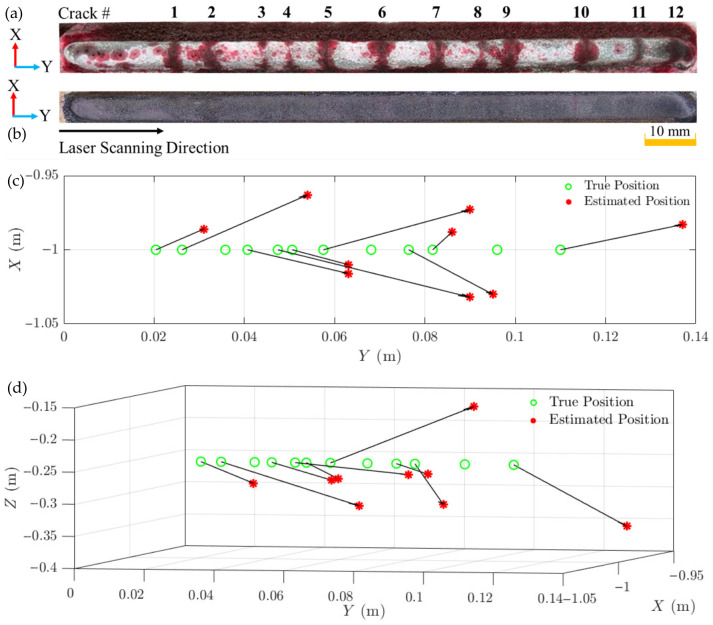
Crack localisation in DED-LB/M-fabricated Track 1 (6th layer). (**a**) Dye penetrant test results on the as-cladded sample, with crack events marked by numbers. (**b**) Top surface image of the as-cladded sample, showing the cladding direction from left to right. (**c**) Comparison of true source positions (green circles) and estimated source locations (red asterisks) in the X–Y plane for identified crack events. (**d**) 3D visualisation comparing true source positions (green circles) and estimated source locations (red asterisks) for identified crack events.

The ASL method encountered significant challenges in accurately estimating the positions of Cracks 3, 8, and 11. In these outlier cases, the estimated TDOA values differed from neighbouring crack events by approximately an order of magnitude, indicating that the GCC-PHAT peak had locked onto process noise components rather than the true crack emission. Consequently, the corresponding source estimates fell outside the physical build envelope or behind the microphone plane and were therefore excluded from Figure 16b,c based on these physical plausibility constraints. These outliers exhibited substantial deviations from their true positions, with errors ranging from centimetres to metres across different dimensions and would otherwise have skewed the overall visualisation and obscured the performance of the more accurately localised cracks. Crack 3’s estimated position (−0.783, 1.762, −0.183 m) deviated substantially from its true position (−1, 0.036, −0.25 m), with a particularly large error in the Y-direction. Crack 8 showed an extreme deviation in all directions, with an estimated position of (0.110, 4.083, −1.927) m compared to its true position of (−1, 0.068, −0.25) m. Similarly, Crack 11s estimated position (0.000, −0.021, 0.0108 m) differed significantly from its true position (−1, 0.096, −0.25 m), particularly in the X- and Z-directions. These significant discrepancies can be attributed to the calculation of incorrect TDOA values, which resulted in the ASL algorithm producing estimates with significant errors. For instance, for Crack 1, TDOA_13_ was estimated at 9.77 × 10^−6^ s, while for Crack 3, TDOA_13_ was estimated at −8.11 × 10^−5^ s. This significant difference in TDOA values implies substantial errors in crack location calculations, highlighting the sensitivity of the localisation algorithm to small variations in time difference measurements. Even small errors in TDOA estimation can propagate and lead to substantial discrepancies in the final estimated crack positions, as evidenced by the large deviations observed for Cracks 3, 8, and 11. The complex acoustic environment inherent to the DED-LB/M process significantly contributes to the observed errors in TDOA measurements and subsequent crack localisation. Several factors inherent to the DED-LB/M process create a challenging environment for acoustic monitoring. Initially, the process generates a highly dynamic and noisy acoustic field, characterised by rapid fluctuations in sound intensity and frequency. Subsequently, the evolving geometry of the deposited material during the DED-LB/M process leads to multiple reflections and refractions of acoustic waves. These phenomena result in complex wave propagation patterns that can significantly change TDOA measurements. Additionally, the current microphone array configuration may possess insufficient spatial resolution or inadequate frequency response characteristics to fully capture the intricate acoustic emissions produced in this environment.

#### 5.1.2. Track 2

**Figure 17:**
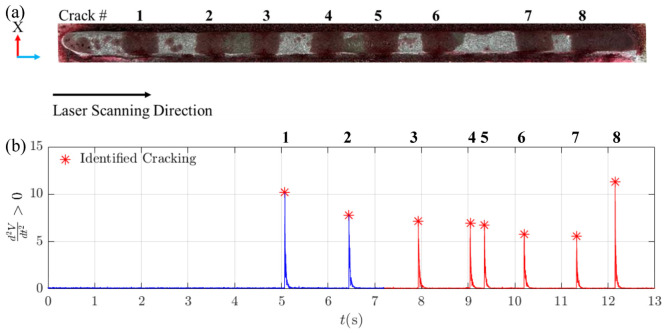
Crack detection in DED-LB/M-fabricated Track 2. (**a**) Dye penetrant test results on the as-cladded sample, with crack marked by numbers. (**b**) Processed acoustic signal from the 6th layer of Track 2, showing identified cracking events marked by red asterisks.

For Track 2, the acoustic signal analysis revealed a different pattern of crack formation compared to Track 1. A total of eight crack events were identified during and just after the fabrication of the sixth layer, as shown in Figure 17b. The occurrence times (*t_c_*) for these cracks were recorded at 5.07 s, 6.44 s, 7.93 s, 9.04 s, 9.35 s, 10.19 s, 11.32 s, and 12.14 s (marked by red asterisks). To validate these findings, a dye penetrant test was conducted, confirming a similar number of cracks at various positions along Track 2 (Figure 17a).

**Figure 18:**
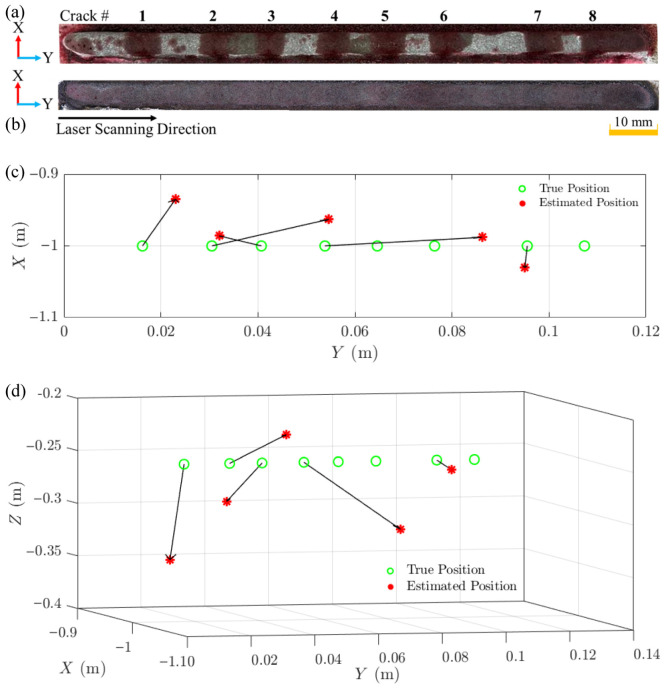
Crack localisation in DED-LB/M-fabricated Track 2 (6th layer). (**a**) Dye penetrant test results on the as-cladded sample, with crack events marked by numbers. (**b**) Top surface image of the as-cladded sample, showing the cladding direction from left to right. (**c**) Comparison of true source positions (green circles) and estimated source locations (red asterisks) in the X–Y plane for identified crack events. (**d**) 3D visualisation comparing true source positions (green circles) and estimated source locations (red asterisks) for identified crack events.

Using the Olympus DSX1000 Digital Microscope, the true crack locations were precisely measured at various distances from the track’s start point, as shown in Figure 18. These measurements translate to the true positions shown in Table 7. The time-windowing approach and TDOA calculation process remained consistent with those used for Track 1. The estimated position of each crack was determined as shown in Table 7. The analysis of the results comparing the true crack positions with the estimated crack positions for Track 2 reveals similar capabilities and limitations of the ASL technique as observed in Track 1. In the X-direction, the technique showed a wider range of errors compared to Track 1, with deviations spanning from 12 mm to 68 mm. The Y-direction estimations exhibited a broader spectrum of accuracy, with errors ranging from 0.7 mm to 32 mm. Z-direction estimates displayed a similar range of errors to Track 1, varying between 5 mm and 100 mm. This consistent Z-direction error range across both tracks indicates that the current array configuration provides reliable centimetre-scale localisation in the horizontal plane but faces persistent challenges in accurately resolving crack depth. From an industrial standpoint, the system should therefore be interpreted primarily as an in-plane diagnostic tool: it can robustly indicate where along the track cracks form and support targeted inspection or process adjustment, whereas precise depth quantification will require future array redesign with increased vertical aperture and additional microphones.

The varying degrees of accuracy observed in the X-, Y-, and Z-directions for Track 2, as well as the significant challenges encountered in accurately estimating the positions of Cracks 5, 6, and 8, can be primarily attributed to the incorrect estimation of the TDOA values. These errors in TDOA estimation are fundamental to the observed discrepancies in crack localisation. The ASL technique relies heavily on precise TDOA calculations to determine crack positions accurately. When the TDOA values are incorrectly estimated, even by small margins, these errors propagate through the localisation algorithm, resulting in substantial deviations in the final estimated crack positions. This is particularly evident in the cases of Cracks 5, 6, and 8, where the errors ranged from centimetres to metres across different dimensions.

The TDOA estimation errors observed in Track 1 are fundamental to the discrepancies in crack localisation accuracy during the DED-LB/M process. Although the GCC-PHAT algorithm is widely regarded as robust in many acoustic localisation applications, its performance is challenged within the DED-LB/M environment, particularly during single-track fabrication. The principal source of localisation error in the present setup is background process noise, which reduces the effective signal-to-noise ratio of crack emissions and can cause the GCC-PHAT peak to lock onto non-crack features in the signal. In this setting, background noise originating from laser–material interaction, powder flow, shielding gas, robotic motion, and ancillary machinery produces a strong broadband acoustic field, especially within the 0.5–6 kHz range, which further broadens or distorts the GCC-PHAT correlation peak.

**Figure 19:**
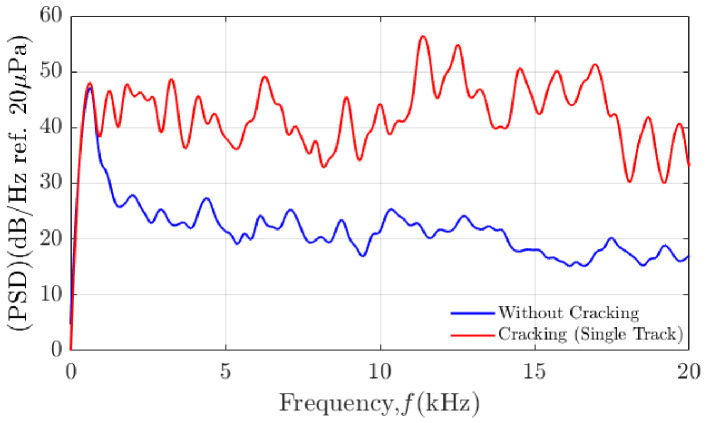
Power Spectral Density (PSD) analysis demonstrating the signal-to-noise ratio (SNR) in DED-LB/M processes with and without cracking events.

A Power Spectral Density (PSD) analysis was conducted to examine the signal-to-noise ratio (SNR) characteristics of the acoustic signals with and without cracking events, as shown in Figure 19. The PSD comparison demonstrates that crack occurrences introduce additional spectral energy components compared to normal depositions. However, the spectral distinction between cracking and non-cracking states is not always pronounced. In particular, dominant process emissions are observed within the 0.5–6 kHz frequency range, which persists both during normal deposition and crack formation. This overlap in frequency content reduces spectral contrast and complicates the reliable extraction of crack-specific acoustic features. In addition, industrial installation constraints (safety clearances and restricted mounting locations) prevent the use of larger or more symmetric 3D microphone arrays, limiting the available aperture and making the localisation performance more sensitive to these noise and timing effects.

The GCC-PHAT algorithm is inherently sensitive to SNR conditions. When crack-generated acoustic emissions do not exhibit sufficient amplitude dominance over background noise, the cross-correlation peak becomes broadened or distorted, leading to increased uncertainty in TDOA estimation [31]. The PSD results in Figure 19 highlight this limitation by showing that crack signals do not always produce a clearly isolated frequency band separate from process noise. Consequently, even small perturbations in delay estimation can propagate nonlinearly into substantial spatial localisation errors.

Furthermore, the DED-LB/M process generates a highly dynamic acoustic field characterised by rapid thermal gradients, evolving build geometry, and multiple reflections from both the substrate and previously deposited layers. These factors alter acoustic wave propagation paths and introduce additional timing uncertainties. Since crack events produce microsecond-scale time delays, exceptionally high temporal precision is required for accurate multilateration. The current four-microphone array configuration provides limited spatial aperture, particularly affecting Z-direction resolution and amplifying the impact of small TDOA inaccuracies.

However, when considering the observed TDOA sensitivity and SNR limitations, the present acoustic localisation framework should be regarded as a promising yet preliminary approach. Improvements in sensor placement, array geometry, microphone count, and advanced signal-processing strategies will be necessary to enhance robustness and precision under industrial operating conditions.

## 6. Conclusions

This study evaluated the application of airborne ASL for crack investigation during the DED-LB/M process under industrial processing conditions. A four-microphone array combined with a GCC-PHAT-based TDOA estimation framework and nonlinear multilateration was implemented to detect and spatially localise crack events during fabrication of multi-layer single tracks.

This research can be summarised by highlighting its primary contributions, limitations, and future directions as follows:

- Crack events were reliably detected acoustically, and spatial localisation in the horizontal plane (X–Y) was achieved with centimetre-scale accuracy under favourable signal-to-noise ratio (SNR) conditions. For Track 1, localisation errors ranged from 12 to 32 mm in the X-direction and 4–27 mm in the Y-direction for most detectable cracks, and Track 2 exhibited similar behaviour with slightly larger variability in the X-direction (12–68 mm). These findings indicate that the current microphone configuration provides reasonable planar localisation capability, whereas depth (Z-direction) estimation consistently showed greater uncertainty, with errors ranging from 5 to 100 mm, and vertical resolution remains a significant limitation.
- Although several crack events were localised with good agreement to post-process microscopic measurements, some cases exhibited significant deviations, highlighting the sensitivity of the localisation framework to timing accuracy and the complex acoustic environment in industrial DED-LB/M. Nevertheless, the overall spatial trends observed in the results support the hypothesis that cracks predominantly form in regions behind the laser beam where rapid solidification and thermal contraction occur. For industrial DED applications, this means the proposed ASL system is currently best suited to flagging crack-prone regions along the track in the horizontal plane, while accurate crack depth estimation will require further optimisation of array geometry and sensor placement.
- The present work establishes the practical feasibility of airborne ASL for crack localisation in DED-LB/M under industrial conditions and demonstrates its potential for real-time, non-contact defect monitoring in large-scale metal AM. At the same time, the current airborne microphone configuration is particularly well-suited to open, large-envelope processes such as laser-based or wire-arc DED, where there is direct acoustic access to the melt pool and track. Transferring the same approach to enclosed laser powder bed fusion (LPBF) systems is expected to be considerably more challenging, as it would require integration of sensors into or onto the build chamber, careful management of internal acoustic reflections and damping, and a full re-optimisation of array geometry and signal-processing parameters for the LPBF acoustic environment. For these reasons, the proposed ASL framework is considered most immediately applicable to DED-class processes that are increasingly used for large-scale AM, whereas extension to LPBF should be viewed as a longer-term adaptation that will demand additional hardware integration and process-specific validation.
- Future research should focus on (i) enhancing spatial resolution, particularly in the Z-direction, through redesigned array geometries and increased microphone count; (ii) improving system robustness by developing more advanced and noise-tolerant TDOA estimation and signal-processing strategies; and (iii) integrating the localisation capability into a comprehensive in situ process-monitoring framework for industrial deployment and extension to other AM processes.

## Figures and Tables

**Table 1:** Microphone positions for acoustic source localisation experiment.

Microphone	X (m)	Y (m)	Z (m)
Mic. #1	1	0	0.45
Mic. #2	1	−0.3, −0.2, …, 0.3 (in 0.1 m increments)	0.3, 0.4, 0.5, 0.6
Mic. #3	1	−0.3, −0.2, …, 0.3 (in 0.1 m increments)	0.3, 0.4, 0.5, 0.6
Mic. #4	1.1	−0.3, −0.2, …, 0.3 (in 0.1 m increments)	0.3, 0.4, 0.5, 0.6

**Table 2:** Optimised microphone array configurations and corresponding source regions.

Array Number	Microphone Positions (m)				Source Region (cm^3^)
	Mic. #1 (X, Y, Z)	Mic. #2 (X, Y, Z)	Mic. #3 (X, Y, Z)	Mic. #4 (X, Y, Z)	
1	(1, 0, 0.45)	(1, 0.3, 0.3)	(1, −0.3, 0.3)	(1.1, −0.1, 0.6)	76.7
2	(1, 0, 0.45)	(1, 0.3, 0.3)	(1, −0.3, 0.3)	(1.1,0.15,0.6)	82.4
3	(1, 0, 0.45)	(1, 0.3, 0.3)	(1, −0.3, 0.3)	(1.1, 0.05, 0.6)	83.5

**Table 3:** Process parameters utilised for the fabrication of Track #1 and Track #2 by using DED-LB/M process.

Laser Power, *P* (W)	Laser Spot Diameter, *d* (mm)	Scanning Speed, *v* mm/min	Powder Feed Rate [g/min]	Total Deposition Time, *t* s	Deposited Track Length, *L* mm
4000	4.8	1000	100	7.2	120

**Table 4:** True positions of cracks in the fabricated structure.

Crack Number	X (m)	Y (m)	Z (m)
1	−1	0.020	−0.25
2	−1	0.026	−0.25
3	−1	0.026	−0.25
4	−1	0.041	−0.25
5	−1	0.047	−0.25
6	−1	0.051	−0.25
7	−1	0.058	−0.25
8	−1	0.068	−0.25
9	−1	0.076	−0.25
10	−1	0.082	−0.25
11	−1	0.096	−0.25
12	−1	0.110	−0.25

**Table 5:** TDOA values for Cracks 1 and 3.

Crack Number	TDOA_13_ (s)	TDOA_14_ (s)
1	0.000009765625	0.000439453125
3	−0.0000810546875	0.000439453125

**Table 6:** Estimated positions of cracks in the fabricated structure.

Crack Number	X (m)	Y (m)	Z (m)
1	−0.986	0.031	−0.288
2	−0.963	0.054	−0.33
3	−0.783	1.762	−0.183
4	−1.016	0.063	−0.271
5	−1.032	0.09	−0.255
6	−1.01	0.063	−0.271
7	−0.973	0.09	−0.17
8	0.11	4.083	−1.927
9	−1.03	0.095	−0.255
10	−0.988	0.086	−0.317
11	0	−0.021	0.0108
12	−0.983	0.137	−0.35

**Table 7:** True and estimated crack positions for Track #2.

Crack Number	True Position (m)	Estimated Position (m)
1	(−1, 0.0162, −0.25)	(−0.935, 0.023, −0.35)
2	(−1, 0.0305, −0.25)	(−0.963, 0.0546, −0.229)
3	(−1, 0.0407, −0.25)	(−0.986, 0.032, −0.288)
4	(−1, 0.0538, −0.25)	(−0.988, 0.0862, −0.317)
5	(−1, 0.0646, −0.25)	(−1.068, 0.303, −28.9)
6	(−1, 0.0764, −0.25)	(0.1432, 3.4, −1.85)
7	(−1, 0.0955, −0.25)	(−1.030, 0.095, −0.255)
8	(−1, 0.1073, −0.25)	(−0.49, 0.074, −0.52)

## Data Availability

The original contributions presented in this study are included in the article. Further inquiries can be directed to the corresponding author.
